# Crushing Behaviors and Energy Absorption Evaluation Methods of Hexagonal Steel Tubular Columns with Triangular Cells

**DOI:** 10.3390/ma15113910

**Published:** 2022-05-31

**Authors:** Weiwei Li, Zhaohui Li, Suhang Li, Peng Wang

**Affiliations:** 1 Department of Civil Engineering, Nanyang Institute of Technology, Nanyang 473004, China; lzh8081@163.com (Z.L.); lsh595423489@163.com (S.L.); 2 Army Engineering University of PLA, Nanjing 210007, China

**Keywords:** hexagonal multi-cell tube, mean crushing force, energy absorption

## Abstract

Under axial compression, multi-cell tubes are considered more effective than single-cell tubes. Regular hexagonal multi-cell tubes (HMT) were designed, tested, and analyzed by finite element modeling (FEM). The crushing mechanism of the HMT was revealed by compression testing and FEM. Experiments and FEM revealed that the mean crushing force of the HMT can be increased by 14% by adopting multi-cell topology, which shortens the folding wavelength and enables HMT progressive crushing. Thus, the HMT is more efficient in energy absorption compared with the conventional regular hexagonal thin-walled tube (HST). More triangular cells result in HMTs with much greater mean crushing force and specific energy absorption. Three evaluation methods were proposed and discussed to determine the effective crushing distance. A plastic model established according to classical simplified super-folding elements was shown to consistently predict the mean crushing force of the HMTs.

## 1. Introduction

Thin-walled structures have been widely applied in the collision kinetic energy dissipation system of almost all transportation vehicles, such as ships [1], trains [2], and aircrafts [3]. In a collision event, thin-walled structures can dissipate a large amount of impact energy through plastic deformation or fracture [4].

As lightweight design requirements in most modern vehicles have progressed, it has become unfeasible to improve the crash resistance of thin-walled tubes simply by increasing the amount of material. Finding methods for designing more lightweight and efficient thin-walled energy absorption devices has become a worldwide pursuit of designers and researchers. For this reason, many researchers have innovatively transformed the original single-cell tubular structures into multi-cell tubular structures. Tang et al. [5] studied the multi-cell square column to improve its energy absorption characteristics and found that the wall thickness and cell number had significant effects on the energy absorption. Chen et al. [6] studied the energy absorption characteristics of foam-filled single-cell, two-cell, and three-cell thin-walled structures by using a finite element method and theoretical calculations; the results showed that the contribution of foam to the energy absorption characteristics of the multi-cell structure could be up to 180%. Qi et al. [7] combined the characteristics of multi-cell tubes and conical tubes and carried out an optimization design of multi-cell taper square tubes under oblique impact. Compared with triangular single-cell tubes, Hong et al. [8] revealed that triangular multi-cell tubes have similar peak force (PF), but the mean crushing force (MCF) would be much larger, indicating that multi-cell structures have greater weight efficiency in energy absorption. Liu et al. [9] studied four kinds of CFRP multi-cell thin-walled tubes with different cell numbers and wall thickness. It was found that the insufficient deformation of t-shaped regions of double-cell CFRP tubes resulted in lower energy absorption than single-cell CFRP tubes. Tran et al. [10] optimized the windowing design of multi-cell thin-walled tubes. Although the initial peak force of the structure was reduced, the energy absorbed by the thin-walled tube with windows was less than that of the traditional tube. Liu et al. [11] studied thin-walled tubes filled with a gradient lattice structure in a multi-cell structure and found that the SEA of the mixed multicellular structure increased by 78.6% over the sum of its individual constituents.

These novel energy-absorbing structures have brought great challenges to theoretical investigation. In order to solve this problem, Chen et al. [6] proposed a simplified super-folding element (SSFE) based on the traditional theory suggested by Abramowicz et al. [12] and Alexander et al. [13]. On this basis, Kim et al. [14] studied the MCF of four-cell structures and proposed a theoretical model for these four-cell structures. Based on the SSFE theory, Zhang et al. [15] theoretically predicted the energy absorption of multi-cell square tubes and predicted the MCF. To improve the energy absorption of square tubes, Nia et al. [16] designed nine-cell square tubes and suggested theoretical formulae to predict the MCF. Zhang et al. [17] established a theoretical model for two crushing modes of a triangular element at any included angle. According to their theory, the membrane energy should vary with the width, thickness, and angle of the wall. However, most of the research has focused on the theoretical and numerical aspects. There are few experimental studies to verify these models.

In this paper, multi-cell hexagonal tubes were designed. The crushing behaviors were investigated experimentally, theoretically, and numerically. On the basis of the SSFE, a theoretical model to predict the MCF was proposed and extended to multi-cell tubes with different cell numbers.

## 2. Hexagonal Multi-Cell Tubular Structures

**Figure 1:**
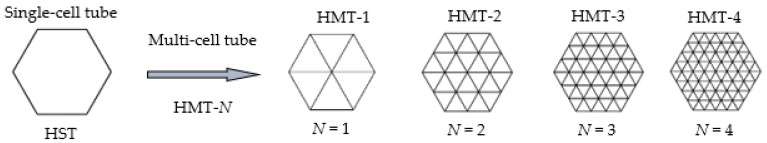
Topology of HMTs with different side segments.

Circular and square sections are the most common shape in the energy absorption field, but Tran et al. [18] thought that corner elements may be superior for absorbing energy. The energy-absorbing ability of conventional regular hexagonal thin-walled tube (HST) is restricted by the long folding wavelength; therefore, by combining regular hexagonal cross section and multi-cell topology, HMTs were designed, made, and tested to improve energy absorption, as shown in Figure 1. The multi-cell structure is of a form such that the sides of the regular hexagon are divided into *N* equal parts by thin ribs, so $6N^{2}$ equilateral triangular cells are formed in the cross section. The number of sub-sides of the HMT, *n*, is given by

(1) $$n=\{\begin{array}{cc} \left(3N+2\right)\times 2+2N & N=1\\ \left(3N+2+3(N+1)+2\right)\times 2+2N & N=2\\ \left(3N+2+3(N+1)+2+3\left(N+2\right)+2\right)\times 2+2N & N=3\\ \ldots \ldots & \ldots \ldots \end{array}\}=9N^{2}+3N$$

Thus, the wall thickness of the HMT, $t_{HMT}$, can be deduced from the wall thickness of the HST, $t_{HST}$, and given by

(2) $$t_{HMT}=\frac{6Nt_{HST}}{n}=\frac{2t_{HST}}{3N+1}$$

According to the principle of equal cross-sectional area and equal quality, the cross-section information of the HST and the HMTs is listed in Table 1.

The tube length is 100 mm, and the side length of the hexagonal column is 60 mm. Each side is divided into *N* segments. As *N* increases, the wall thickness of the HMT becomes smaller. For example, compared to the ST, whose walls are 3 mm thick, the solid wall thickness is reduced to only 0.375 mm. Besides, all the solid components have the same cross-sectional area of 1080 mm^2^.

**Figure 2:**
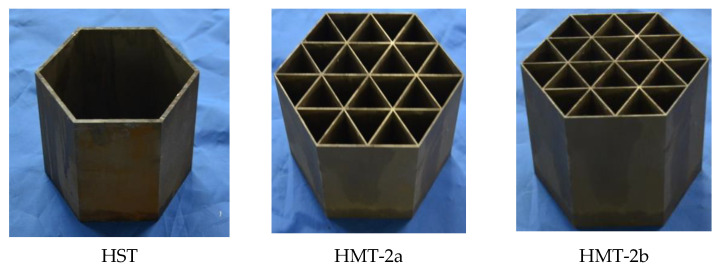
Manufactured thin-walled steel hexagonal tubes.

Accordingly, one HST and two HMT-2s were designed and made by the line-cutting process with steel Q235, as shown in Figure 2.

**Figure 3:**
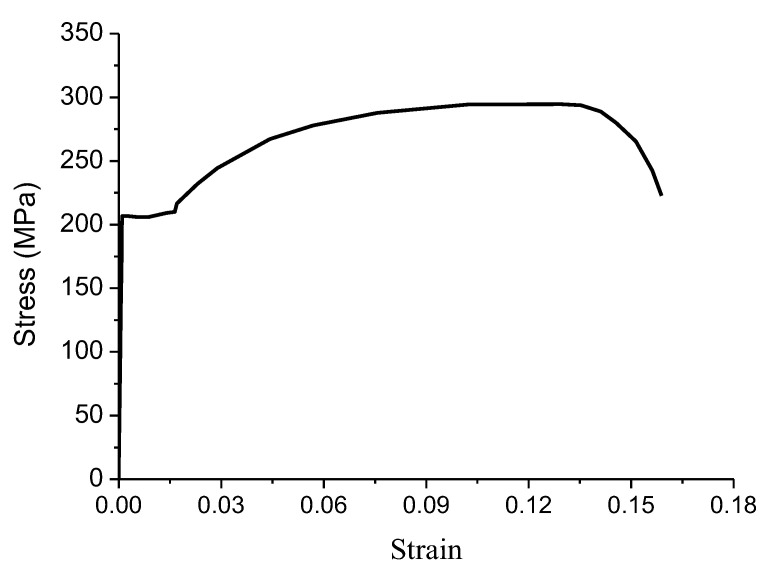
Engineering stress–strain curve of steel Q235 [19].

The stress–strain curve of steel Q235 is shown in Figure 3 and listed in Table 2 [19]. The plastic stress is the average of the initial yield stress and the ultimate stress.

## 3. Experimental Behaviors

### 3.1. Crushing Behaviors

**Figure 4:**
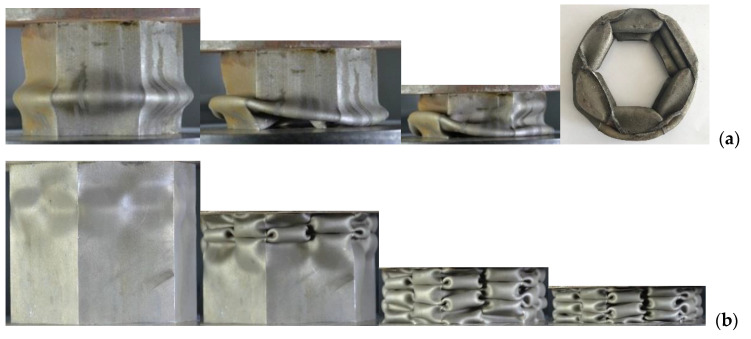

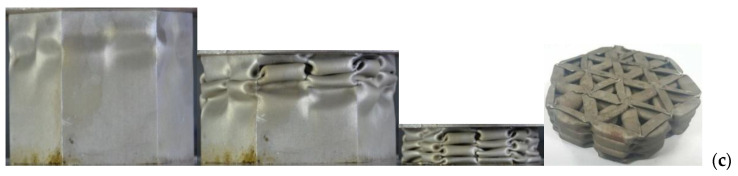
Crushing patterns of (**a**) hexagonal HST [19], (**b**) HMT-2a, and (**c**) HMT-2c.

The thin-walled tubes were compressed at a loading rate of 2.0 mm/min on a 600 kN universal test machine. The crushing process is displayed in Figure 4. For the hexagonal ST, the angular element was fully expanded, resulting in two relatively complete folding waves, as shown in Figure 4a [19]. In the case of the MTs, the cell dimension was greatly reduced, and the folding wavelength can be greatly shortened. As shown in Figure 4b,c, the HMTs have over four folding waves.

**Figure 5:**
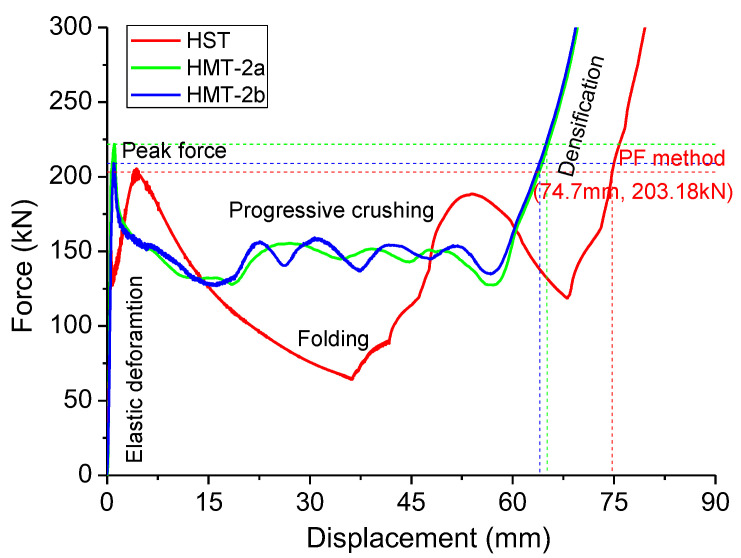
Force–displacement curves of compressed hexagonal HST and HMTs.

Shorter folding wavelength results in more stable deformation curves, as shown in Figure 5, where the force–displacement curves are displayed. The displacement curve of the HST has great fluctuations with two waves, while the HMTs have more stable deformations with four waves. The fluctuation amplitude depends on the number of folding waves. Obviously, the amplitude of displacement oscillation of the ST is much larger, which will weaken its energy absorption.

### 3.2. Evaluation Methods

**Figure 6:**
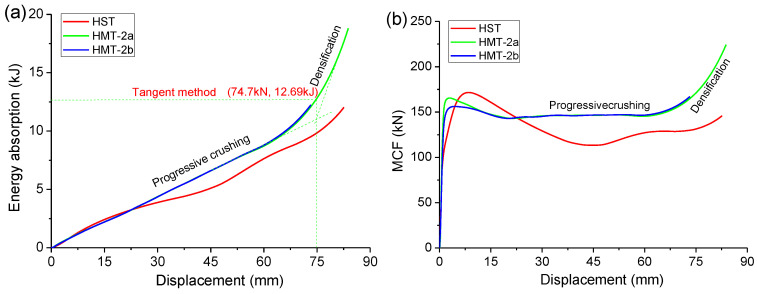
(**a**) Energy absorption and (**b**) MCF of compressed hexagonal HST and HMTs.

As shown in Figure 6a, the HMTs have much greater energy absorption than the ST. The energy absorption curves remain quasi-linear and the densification stage is not well-identified. In densification, the slope increases.

**Figure 7:**
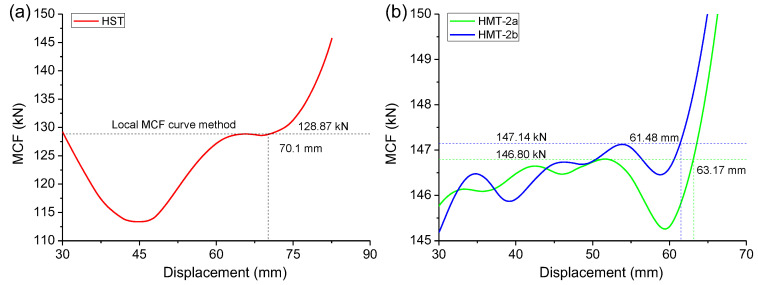
Local MCF curve of (**a**) HST and (**b**) HMTs to determine the MCF and the effective crushing distance.

The HMTs also have much greater MCF than the HST, as shown in Figure 6b. The MCF curves clearly embody the three stages of deformation. In progressive crushing, the MCF changes little. In densification, the MCF increases rapidly. To determine the MCF and the effective crushing distance, local MCF curves were adopted, as shown in Figure 7. The MCF curve rises dramatically in densification. Before densification, the nearest local peak value is selected as the effective MCF of the tube, and this value in densification is selected to determine effective crushing distance.

As listed in Table 3, the MCF of ST is 128.7 kN, but its effective crushing distance is 70.1 mm. The HMTs have greater MCF, 146.8 kN and 147.1 kN, respectively, but they have a little shorter effective crushing distance, 63.17 mm and 61.48 mm, respectively. When the local MCF Curve method is adopted, the mean crushing force of HMT-2 increases by 14% compared with that of HST.

Three methods can be applied to decide the effective crushing distance, including the PF method, the tangent method, and the local MCF curve method, as shown in Figure 5, Figure 6 and Figure 7. As these methods will suggest different effective crushing distance, the MCF and energy absorption will differ greatly, as listed in Table 4. The tangent method suggests much greater effective crushing distance and MCF, while the other two methods present comparable data.

## 4. Theoretical Analysis

The energy absorption of progressive folding is composed of the deformation energy of the cell membrane and the bending deformation of the plastic hinge. According to the conservation of the energy, the work supplied by the MCF is equal to the energy absorbed by the plastic deformations of the membrane extension and the plastic hinge bending, which is given by

(3) $$2HP_{m}\kappa =E_{b}+E_{m}$$

where $E_{b}$, $E_{m}$ and $P_{m}$ denote the bending energy, the membrane energy, and the MCF, respectively. *H* denotes the half wavelength of the folding and κ denotes the effective crushing distance coefficient, defined by the ratio of the effective crushing distance to the tube length.

**Figure 8:**
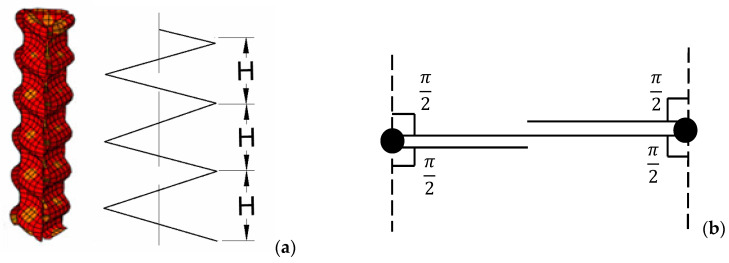
Plastic models of part folding: (**a**) cell folding and (**b**) bending hinge.

As shown in Figure 8, the bending energy consumed by each panel is equal to the sum of the energy absorbed by the plastic bending hinge and given by [19]

(4) $$E_{b}=\sum_{i=1}^{6}\theta_{i}M_{0}L=\frac{1}{2}\pi \sigma_{0}tS$$

where θ=2π is the rotation angle at the bending hinge line, $M_{0}=\frac{1}{4}\sigma_{0}t^{2}$ is the fully plastic bending moment, L is the total length of sectional width, t denotes the wall thickness of the thin-walled plate, S denotes the cross-section area of solid walls with steel materials, and $\sigma_{0}$ denotes the flow stress, which is approximately the average of the yield stress and ultimate stress.

**Figure 9:**
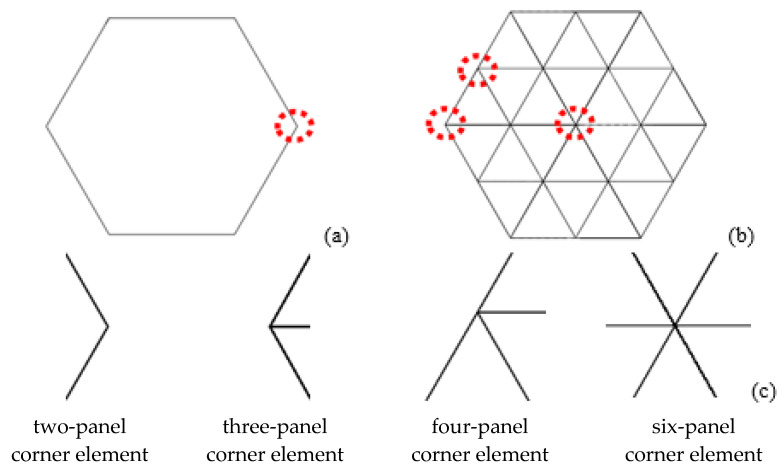
Cross section of (**a**) HST and (**b**) HMT and (**c**) typical angular elements.

In order to analyze the plastic deformation of the corner elements, four basic elements including two-panel, three-panel, four-panel, and six-panel corner elements were analyzed, as shown in Figure 9.

The membrane energy can be obtained by integrating stretching or compression area:

(5) $$E_{m}=\int_{s}\sigma_{o}t_{i}ds_{i}=\sum_{i=1}^{j}\Delta S_{i}t_{i}\sigma_{0}$$

According to the finite element calculation method of Li et al. [19], the membrane energy is calculated by

(6) $$E_{m}^{2p}=\sum_{i=1}^{n}\Delta S_{i}t_{i}\sigma_{0}=\sqrt{3}H^{2}t\sigma_{0}$$

for two-panel corner elements,

(7) $$E_{m}^{3p}=\sum_{i=1}^{n}\Delta S_{i}t_{i}\sigma_{0}=3\sqrt{3}H^{2}t\sigma_{0}$$

for three-panel corner elements, and

(8) $$E_{m}^{4p}=\sum_{i=1}^{n}\Delta S_{i}t_{i}\sigma_{0}=4\sqrt{3}H^{2}t\sigma_{0}$$

for four-panel corner elements. According to Sun’s method [20], for the six-panel corner element the membrane energy, $E_{m}^{6p}$, is calculated by

(9) $$E_{m}^{6p}=\sum_{i=1}^{n}\Delta S_{i}t_{i}\sigma_{0}=6\sqrt{3}H^{2}t\sigma_{0}$$

Then the MCF of thin-walled structures can be calculated by

(10) $$P_{m}=\frac{E_{b}+E_{m}}{2H\kappa }=\frac{\frac{1}{2}\pi \sigma_{0}tS+6E_{m}^{2p}}{2H\kappa }$$

and

(11) $$P_{m}=\left(\frac{\pi S}{4H}+3\sqrt{3}H\right)\frac{\sigma_{0}t}{\kappa }$$

for the HST, and

(12) $$P_{m}=\frac{E_{b}+E_{m}}{2H\kappa }=\frac{\frac{1}{2}\pi \sigma_{0}tS+6E_{m}^{3p}+6\left(N-1\right)E_{m}^{4p}+\left[N^{3}-{\left(N-1\right)}^{3}\right]E_{m}^{6p}}{2H\kappa },$$

and

(13) $$P_{m}=\frac{\pi \sigma_{0}tS}{4H\kappa }+\frac{9+12\left(N-1\right)+3\left[N^{3}-{\left(N-1\right)}^{3}\right]}{\kappa }\sqrt{3}Ht\sigma_{0},$$

for the HMT. The folding wavelength can by calculated by

(14) $$\frac{\partial P_{m}}{\partial H}=0$$

For the HST,

(15) $$H=\sqrt{\frac{\pi S}{12\sqrt{3}}}$$

and

(16) $$P_{m}=\frac{\sqrt{3\sqrt{3}\pi S}}{\kappa }t\sigma_{0}$$

For the HMT,

(17) $$H=\sqrt{\frac{1}{18+24\left(N-1\right)+6\left[N^{3}-{\left(N-1\right)}^{3}\right]}\frac{\pi S}{2\sqrt{3}}},$$

and

(18) $$P_{m}=\frac{1}{\kappa }\sqrt{9+12\left(N-1\right)+3\left[N^{3}-{\left(N-1\right)}^{3}\right]}\sqrt{\sqrt{3}\pi S}\sigma_{0}t.$$

**Figure 10:**
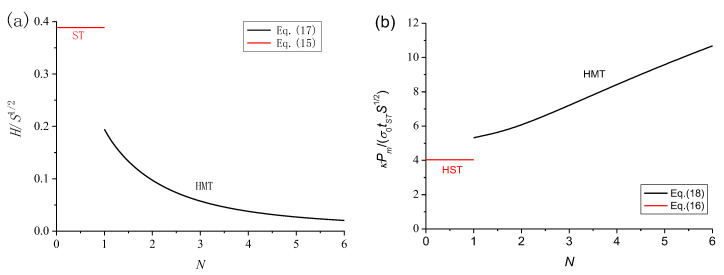
Dimensionless theoretical (**a**) folding wavelength and (**b**) MCF.

Dimensionless theoretical folding wavelength and MCF are compared in Figure 10. Theoretically, the folding wavelength of the HMT is much shorter than that of the HST, as shown in Figure 10a, indicating that the HMT would have much greater MCF and energy absorption, as shown in Figure 10b. Along with the increase of the segment number, *N*, the folding wavelength will be further shortened, as shown in Figure 10a, indicating that the HMT with more micro-cells would have much greater MCF and energy absorption, as shown in Figure 10b.

Then the MCF can be predicted, as listed in Table 5. Compared with the tested data, the predictions are only a little greater and the errors are within 10% and acceptable. The theory can be validated by the experiments.

## 5. FEM Analyses

### 5.1. FEM Method

**Figure 11:**
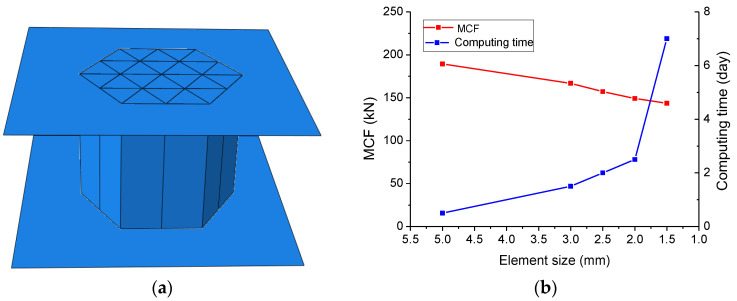
(**a**) Finite element model of tube HMT-2 and (**b**) effect of element size on simulated MCF and computing time.

FEM was applied to extend the investigations on the energy absorption of HMTs from HMT-2 to HMT-5 based on commercial code ABAQUS/Explicit. S4R shell element was adopted to model the solid walls of the HMT, as shown in Figure 11a. The bottom end of the sample was fixed on a rigid plate, and the rigid plate covering the top only produced a downward displacement of 80 mm within 0.01 s. The interaction between the HMT and the rigid plates was modeled by a self-contact method, and the friction effect between the contact surfaces was set as 0.2. After checking the sensitivity of the element size, meshes of 2 mm might be a reasonable choice to balance the accuracy and the calculation time, as shown in Figure 11b.

**Figure 12:**
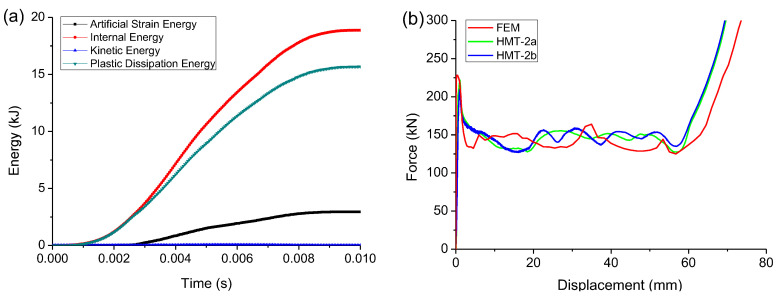
(**a**) Energy variation of HMT-2 in computation to clarify the quasi-static loading condition and (**b**) FEM force–displacements curves compared with the experiment to validate the feasibility of the FEM.

In order to avoid the hourglass problem and ensure no increase of the artificial energy of the system during the calculation, the kinetic energy, the internal energy, the plastic dissipative energy, and the artificial energy of each model system were tested. HMT-2 was taken as an example to calculate the change of the system energy during the crushing. As shown in Figure 12a, the artificial strain energy is always less than 5% of the internal energy of the system, and the hourglass deformation was well-restrained. During the crushing, the kinetic energy is too small to be considered. It approaches zero while the plastic dissipation reaches its maximum value. Therefore, under the preset loading rate the tube is still in quasi-static loading state.

The FEM was firstly applied to simulate the crushing of HMT-2 to check its feasibility. The FEM force–displacement curve of HMT-2 is compared with the tested curves, as shown in Figure 12b. They are consistent. Details of the comparisons are listed in Table 6. The errors between the FEM and the experiments are within 10% and acceptable.

### 5.2. FEM Results

**Figure 13:**
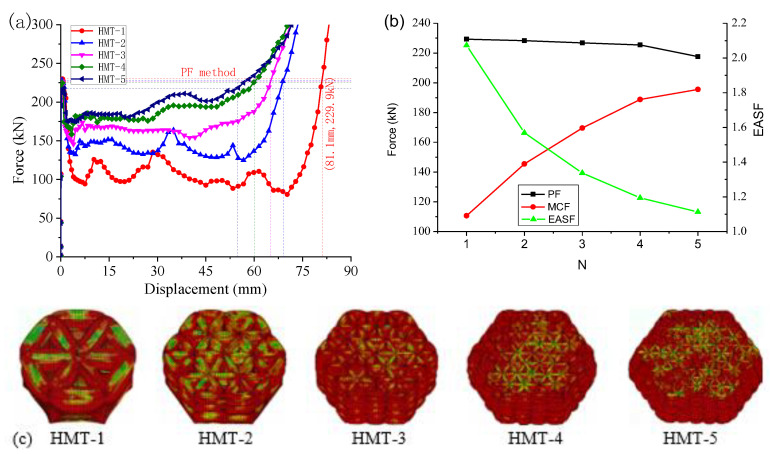
The crushing performance of multi-cell thin wall tube: (**a**) force–displacement curves, (**b**) energy absorption parameters curves, and (**c**) folding modes.

Details of the FEM simulation are displayed in Figure 12. The deformation of the HMTs includes elastic deformation, progressive crushing, and densification, as shown in Figure 13a. All these tubes have comparable PF, while the MCF increases with the side segment number, *N*, as shown Figure 13b, which makes energy absorption stability factor (EASF) gradually tend to 1.0, which is defined by

(19) $$EASF=\frac{P_{\max}}{P_{m}}.$$

As all these tubes have identical mass, it means that the multi-cell topology effectively increases the energy absorption. There are only two complete folds for HMT-1, while there are four for HMT-2. With the increase of *N*, the number of complete folds grows slowly until HMT-5, with six complete folds, as shown in Figure 13c.

**Figure 14:**
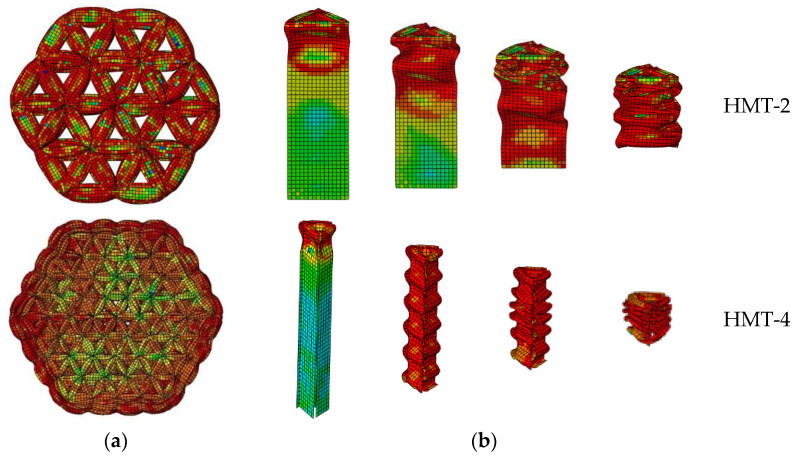
FEM progressive crushing of compressed HMTs: (**a**) top view and (**b**) folding of a typical unit cell column.

In this research, all HMTs have similar progressive crushing, as shown in Figure 14. As N increases, the side length of the unit triangular cell is greatly reduced, which will restrain the local buckling wavelength. Then, the number of folds increases and the folding wavelength is shortened at the same time.

### 5.3. Evaluation Methods

**Figure 15:**
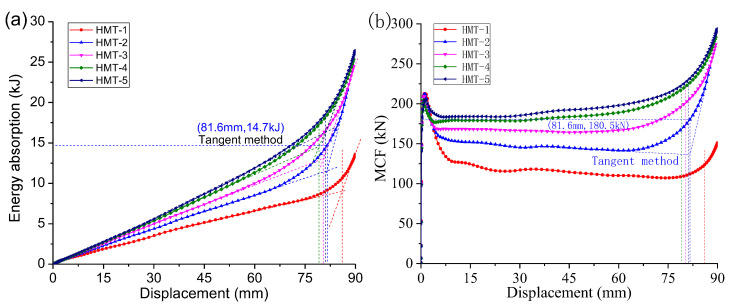
FEM (**a**) energy absorption and (**b**) MCF of compressed HMTs.

The energy absorption and the MCF increase with the side segment number, *N*, as shown in Figure 15. To decide the effective energy absorption, two methods can be considered. As the curves have two quasi-linear segments, their tangent lines will intersect at one point. This point decides the effective crushing distance. A vertical line can be made over this point to intersect with the function at a new point, which will decide the energy absorption or the MCF. According to this method, the effective crushing distance of MT-2 is 81.6 mm. The energy absorption is 14.7 kJ and the MCF is 180.5 kN. For MT structure, the MCF increases with the increase of *N*, as shown in Table 7. As more triangular cells are added to the column, the energy absorption is much greater.

As shown in Figure 13a, a PF-based method is suggested. Applying this method, a point having identical force with the PF is decided at the densification stage. The effective crushing distance of MT-2 is only 69.1 mm. The energy absorption is 10.1 kJ and the MCF is 145.5 kN. All these values are much smaller than those in Table 8. It was also found that the MCF will still increase with *N*; meanwhile, the effective crushing distance is greatly shortened. Adopting this method, HMTs with more cells have little advantage in energy absorption.

**Figure 16:**
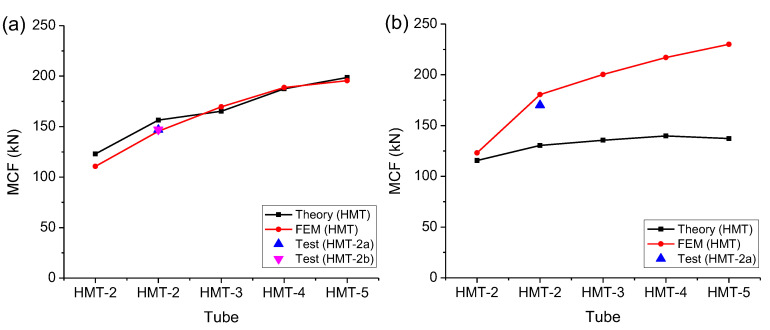
Predicted MCFs based on (**a**) the PF method and (**b**) the tangent method.

The theoretical prediction is almost consistent with the FEM and the experiment, as shown in Figure 16a. The plastic theory is valid. Detailed comparisons are listed in Table 9, where the plastic model can consistently predict the MCF and energy absorption decided by the PF method. The error could be controlled to within 10% in this study.

The tangent method will include part of the energy absorbed in the densification. As the plastic model cannot describe the plastic deformation in the densification, it will underestimate the MCF and energy absorption decided by the tangent method, as shown in Figure 16b and listed in Table 10. As the crushing ratio, *κ*, is much larger in this method, the predicted MCF is even smaller, such that the errors are much greater. In this case, the suggested model is invalid. It is interesting that the FEM and the tested value are still consistent.

## 6. Conclusions

To improve the energy absorption of thin-walled tubes, hexagonal multi-cell tubular structures were designed and manufactured. Their crushing behaviors were investigated by testing, simulating, and analyzing. Through the analyses it can be concluded that:

(1) As revealed by testing, when the local MCF curve method is adopted, the mean crushing force of HMT-2 increases by 14% compared with that of HST. Multi-cell topology effectively shortens the folding wavelength and greatly increases the number of plastic energy absorbing mechanisms.

(2) Three evaluation methods were proposed to determine the effective crushing distance and evaluate the energy absorbing ability, including the PF method, the local MCF curve method, and the tangent method. The values recommended by the first two methods are similar. The last method suggests a much larger value, as part of the densification deformation is included.

(3) A plastic model was proposed to predict the MCF of the HMT. The model can explain the shortening of the folding wavelength with the increase of the number of micro-cells.

(4) As revealed by FEM and theoretical analysis, when the HMT has more micro-cells, the folding wavelength will be further shortened and the MCF and the energy absorption will continue to increase. The error of thin-walled tubes is within 10% and acceptable.

## Figures and Tables

**Table 1:** Dimensions of designed hexagonal thin-walled tubes.

Tube	Side Wall Segment Number, *N*	Cell Number	Cell Dimension, *B* (mm)	Skin Thickness, *t* (mm)	Tube Length, *h* (mm)	Area of Solid Walls, *S* (mm^2^)
HST	1	1	60	3	100	1080
HMT-1	1	6	60	1.5	100	1080
HMT-2	2	24	30	0.857	100	1080
HMT-3	3	54	20	0.6	100	1080
HMT-4	4	96	15	0.462	100	1080
HMT-5	5	150	12	0.375	100	1080

**Table 2:** Material properties of steel Q235 [19].

Property	Symbol	Value
Young’s modulus	E	210 GPa
Initial yield stress	$\sigma_{y}$	206 MPa
Ultimate stress	$\sigma_{u}$	294 MPa
Plastic stress	$\sigma_{0}$	250 MPa
Poisson’s ratio	υ	0.25

**Table 3:** Experimental crushing resistance based on local MCF curve.

Tube	PF (kN)	MCF (kN)	MCF/PF	Effective Crushing Distance (mm)
HST	203.2	128.7	0.633	70.1
HMT-2a	221.9	146.8	0.662	63.17
HMT-2b	208.9	147.1	0.704	61.48

**Table 4:** Comparisons between three methods.

Tube	Local MCF Curve Method		Tangent Method		PF Method	
	MCF (kN)	Effective Crushing Distance (mm)	MCF (kN)	Effective Crushing Distance (mm)	MCF (kN)	Effective Crushing Distance (mm)
HST	128.7	70.1	-	-	131.0	74.7
HMT-2a	146.8	63.17	170.0	74.7	148.6	65.07
HMT-2b	147.1	61.48	-	-	149.0	63.99

**Table 5:** Comparisons between theory and test of MCF based on PF method.

Case	Theory (kN)	Test (kN)	Error (%)
HST	133.3	131.0	1.7
HMT-2a	163.5	148.6	9.1
HMT-2b	163.5	149.0	8.8

**Table 6:** Comparisons between FEM and experiments.

Case	Peak Force (kN)			Mean Crushing Force (kN)		
	FEM	Experiment	Error (%)	FEM	Experiment	Error (%)
HMT-2a	228.3	221.9	2.9	145.5	146.8	−0.9
HMT-2b		208.9	9.3		147.1	−1.1

**Table 7:** FEM-simulated crushing data of HMTs based on tangent method.

Tube	HMT-1	HMT-2	HMT-3	HMT-4	HMT-5
*d* (mm)	86.1	81.6	80.4	79.1	81.1
*P*_max_ (kN)	229.9	228.3	226.8	225.4	217.6
*P_m_* (kN)	123.1	180.5	200.4	217.0	230.1
*P*_max_/P_m_	1.88	1.26	1.13	1.04	0.95
SEA (J/g)	10.6	14.7	16.1	17.2	18.6
*κ*	0.861	0.816	0.804	0.791	0.811

**Table 8:** FEM-simulated crushing data of HMTs based on PF method.

Tube	HMT-1	HMT-2	HMT-3	HMT-4	HMT-5
*d* (mm)	81.1	69.1	65.1	60.1	54.9
*P*_max_ (kN)	229.9	228.3	226.8	225.4	217.6
*P_m_* (kN)	110.6	145.5	169.6	188.8	195.5
*P*_max_/P_m_	2.07	1.57	1.34	1.19	1.11
SEA (J/g)	9.0	10.1	11.1	11.4	10.8
*κ*	0.811	0.691	0.651	0.601	0.549

**Table 9:** Theoretical predicted MCFs for HMTS based on PF method.

Tube	HMT-1	HMT-2	HMT-3	HMT-4	HMT-5
Theory	122.7	153.9	167.5	184.0	202.7
FEM	110.6	145.5	169.6	188.8	195.5
Test	-	148.6 149.0	-	-	
Error	−9.8%	−5.5%	1.3%	2.6%	−3.6%

**Table 10:** Theoretical predicted MCFs for HMTS based on tangent method.

Tube	HMT-1	HMT-2	HMT-3	HMT-4	HMT-5
Theory	115.6	130.4	135.6	139.8	137.2
FEM	123.1	180.5	200.4	217.0	230.1
Test	-	170.0	-	-	-
Error	6.5%	38.4%	47.8%	55.2%	67.7%

## Data Availability

This study did not report any data.
